# Fatigue after Stroke: Baseline Predictors and Influence on Survival. Analysis of Data from UK Patients Recruited in the International Stroke Trial

**DOI:** 10.1371/journal.pone.0016988

**Published:** 2011-03-18

**Authors:** Gillian E. Mead, Catriona Graham, Paul Dorman, Slot Karsten Bruins, Steff C. Lewis, Martin S. Dennis, Peter A. G. Sandercock, on behalf of the UK Collaborators of IST

**Affiliations:** 1 University of Edinburgh, Edinburgh, United Kingdom; 2 Wellcome Trust Clinical Research Facility, University of Edinburgh, Edinburgh, United Kingdom; 3 Department of Neurology, Newcastle Acute Hospitals NHS Foundation Trust, Newcastle-upon-Tyne, United Kingdom; 4 Division of Medicine, Oslo University Hospital-Ullevål, Oslo, Norway; Universidad Peruana Cayetano Heredia, Peru

## Abstract

**Background and Purpose:**

Little is known about the associations of post-stroke fatigue or its influence on survival. The vitality component of the Short Form 36 (SF-36) is a valid and reliable measure of post-stroke fatigue. We sought to identify associates of post-stroke fatigue and determine whether fatigue predicted survival.

**Methods:**

We used SF-36 vitality scores obtained by postal questionnaires from 1080 UK patients randomised in the International Stroke Trial, at a mean of 64 weeks after stroke onset. We used logistic regression to explore factors at randomisation which predicted SF-36 vitality at follow-up, and the relationship between SF-36 vitality and both SF-36 mental health and SF-36 emotional role function at follow-up. We used Cox proportional hazards to explore the influence of SF-36 vitality at follow-up on subsequent survival, using four different statistical models for handling missing data.

**Results:**

Female sex, increasing age, lower mental health and lower emotional role function scores were associated with greater degrees of fatigue after stroke (i.e. lower vitality scores) but these factors explained <30% of the variance (R^2^) in fatigue. In two models, fatigue at follow-up was associated with shorter subsequent survival.

**Conclusion:**

Increasing age, female sex, emotional role function and mental health were associated with increased fatigue at a mean of 64 weeks after stroke onset, but explained less than 30% of the variance. Fatigue was associated with reduced subsequent long-term survival in 2/4 models. Further work is needed to identify the biological substrate of fatigue and to clarify its influence on survival.

## Introduction

Fatigue is common after stroke, with estimates of prevalence ranging from 16% [1] to 70% [2]. Fatigue is one of the most distressing symptoms after stroke [3], yet its aetiology remains uncertain. In the general non-stroke population, fatigue is often a symptom of depression. Several cross-sectional studies [1], [3]-[8] and one longitudinal study [9] have sought associations of fatigue after stroke using different generic fatigue scales. Most [1], [3], [4], [6], [9] but not all [7], [8] have found associations with depression. One previous study found an association with variability in blood pressure [10]. Early studies of fatigue after stroke suggested that it is more common in brain stem lesions [7], but other studies have not shown such an association [1], [4], [6], [8], [9], [11], [12]. The single previous study that investigated associations between fatigue and survival found that it was independent predictor of shorter survival [1].

All previous studies of fatigue after stroke used generic fatigue scales which had not been tested for validity or reliability in stroke. The vitality domain of the Short-Form 36 (SF-36) has face validity and is reliable after stroke [12], and correlates with other fatigue scales [11]. Stroke patients fulfilling a case definition for clinically significant fatigue have lower SF-36 vitality scores than those without clinically significant fatigue [12].

The vitality domain in the original version of the SF-36 includes four questions: In the past four weeks: ‘Did you feel full of pep?’, ‘Did you have a lot of energy?’, ‘Did you feel worn out?’ and ‘Did you feel tired?’ Responses to each question are: ‘All of the time’, ‘Most of the time’, ‘a Good bit of the time’, ‘Some of the time’ ‘A little of the time’ and ‘None of the time’ [13]. After the responses to the individual questions were recorded, a total score for vitality can be calculated according to the SF-36 scoring manual [13]. The range of possible scores is from 0 to 100, with higher scores indicating higher vitality (i.e. less fatigue).

Our aims were to determine: a) what factors at stroke onset predicted SF-36 vitality scores [13] measured a few months later, b) the relationship between SF-36 vitality measured in survivors a few months after stroke and mood (SF-36 mental health domain and emotional role function domain) and c) influence of SF-36 vitality in survivors on subsequent survival.

## Materials and Methods

We used data from patients in the International Stroke Trial (IST), recruited by UK Centres, who had participated in a substudy on quality of life after stroke [14]-[17].

### The International Stroke Trial

The IST was a randomised controlled trial of the effects of aspirin, subcutaneous heparin, both or neither, among 19,435 patients with acute ischaemic stroke, recruited within 48 hours of stroke onset [17]. To receive the patient's randomised treatment allocation, clinicians telephoned a central randomisation service. During this telephone call, after data on age, gender, pathological subtype of stroke, stroke subtype, neurological deficits, blood pressure and cardiac rhythm had been recorded and checked, the system generated the treatment allocation [17].

### Collection of SF-36 data

A subset of the patients recruited by the UK centres in the IST trial participated in a study of quality of life after stroke. Surviving patients (n = 2253) were sent either a SF-36 or EuroQUOL (another scale to measure quality of life) by postal questionnaire [14]-[16] at a mean of 64 (SD 30) weeks following randomisation. Between 2^nd^ March 1993 and 31^st^ May 1995, 1400 patients were sent a SF-36, of whom 1080 responded (77%) [14]-[16]. The SF-36 includes the following domains: vitality, physical functioning, physical role function, social functioning, bodily pain, mental health, emotional role functioning and general health [13]-[16]. Each domain, including the mental health and emotional role function domains, includes several questions. We changed the question ‘Did you feel full of pep?’ to ‘Did you feel full of life?’ to ensure its cultural relevance [13]-[15]. The responses to the questions allow a total score for each domain to be calculated using the standardised SF-36 scoring manual [13]. Scores for each domain range from 0-100, with higher scores indicating better health.

These data allowed us to explore the relationship between SF-36 vitality a few months after stroke, patient characteristics at randomisation and the SF-36 role emotional and SF-36 mental health a few month after stroke.

### Collection of survival data

All UK patients in IST were ‘flagged’ at the Office for National Statistics central registry of deaths, enabling us to collect data on all deaths occurring in this cohort up to November 2000 giving us survival data for between 5 and 7 years after the assessment of SF-36 [18].

### Statistical analysis of factors at randomisation associated with fatigue on follow-up

We analysed fatigue severity (SF-36 vitality) as a continuous variable. We performed multiple linear regression analyses to determine the relationship between fatigue and the following variables measured at randomisation: age, gender, pathological subtype of stroke (ischaemic, haemorrhagic or indeterminate), stroke subtype (total anterior circulation syndrome (TACS), partial anterior circulation syndrome (PACS), lacunar syndrome (LACS), posterior circulation syndrome (POCS) or ‘other’), presence of a visible infarct on brain imaging, cardiac rhythm (atrial fibrillation or sinus rhythm) and systolic blood pressure (<140, 140-159, 160-79, >180mmHg.

Results were reported in the form of regression equations. For a simple linear regression, with one predictor variable, the equation would be of the form Y = a+b*X i.e. the *Y* variable can be expressed in terms of a constant (*a*) and a slope (*b*) multiplied by the *X* variable. For multiple linear regression analysis when there is more than one independent variable (as in this study), the regression line cannot be visualized in the two dimensional space, but can be computed using a linear equation containing all those variables. Multiple regression procedures estimate a linear equation of the form: Y = a+b_1_*X_1_+b_2_*X_2_+…+b_p_*X_p_.

We performed four sensitivity analyses to determine the robustness of the estimates to different assumptions about missing data, in which we: i) removed those with missing vitality data ii) imputed the minimum value recorded iii) imputed the maximum value recorded iv) imputed the mean value observed.

### Statistical analysis of association between mood and fatigue

At follow-up, patients completed the emotional role score and the mental health scores of the SF-36. The responses were used to calculate a total score for each domain [13], ranging from 0-100, with higher score indicating better health.

We produced multiple regression models which included the variables at randomisation shown to have an effect on vitality score (i.e. the final models generated in the previous section) and the SF-36 mental health and SF-36 emotional role scores. Missing scores for emotional role function and mental health were treated in the same way as missing scores for vitality.

### Statistical analysis of the influence of fatigue at follow-up on subsequent survival

We generated a Cox proportional hazards model containing the following variables: age, sex, pathological subtype, stroke subtype, visible infarct, atrial fibrillation (at randomisation), systolic blood pressure (at randomisation) and all component parts of the SF-36 at follow-up (vitality, role-emotional, mental health, physical function, bodily pain, general health and social function) with the exception of role-physical. We did not include role-physical as this measures similar constructs to physical function and there were more missing values for role physical than for physical function. For these analyses, survival was measured from the date on which SF-36 vitality was measured.

Where the initial model generated contained variables which did not reach statistical significance at the 5% level, we removed the least significant variable and generated a new model. We repeated this process until the only variables remaining in the model were those which reached statistical significance. We calculated four different models to explore robustness to differing assumptions about missing variables.

Using the threshold value of at least ‘10 patients per variable’ rule for the analyses of regression, we had sufficient power for our analyses [19].

## Results

**Figure 1:**
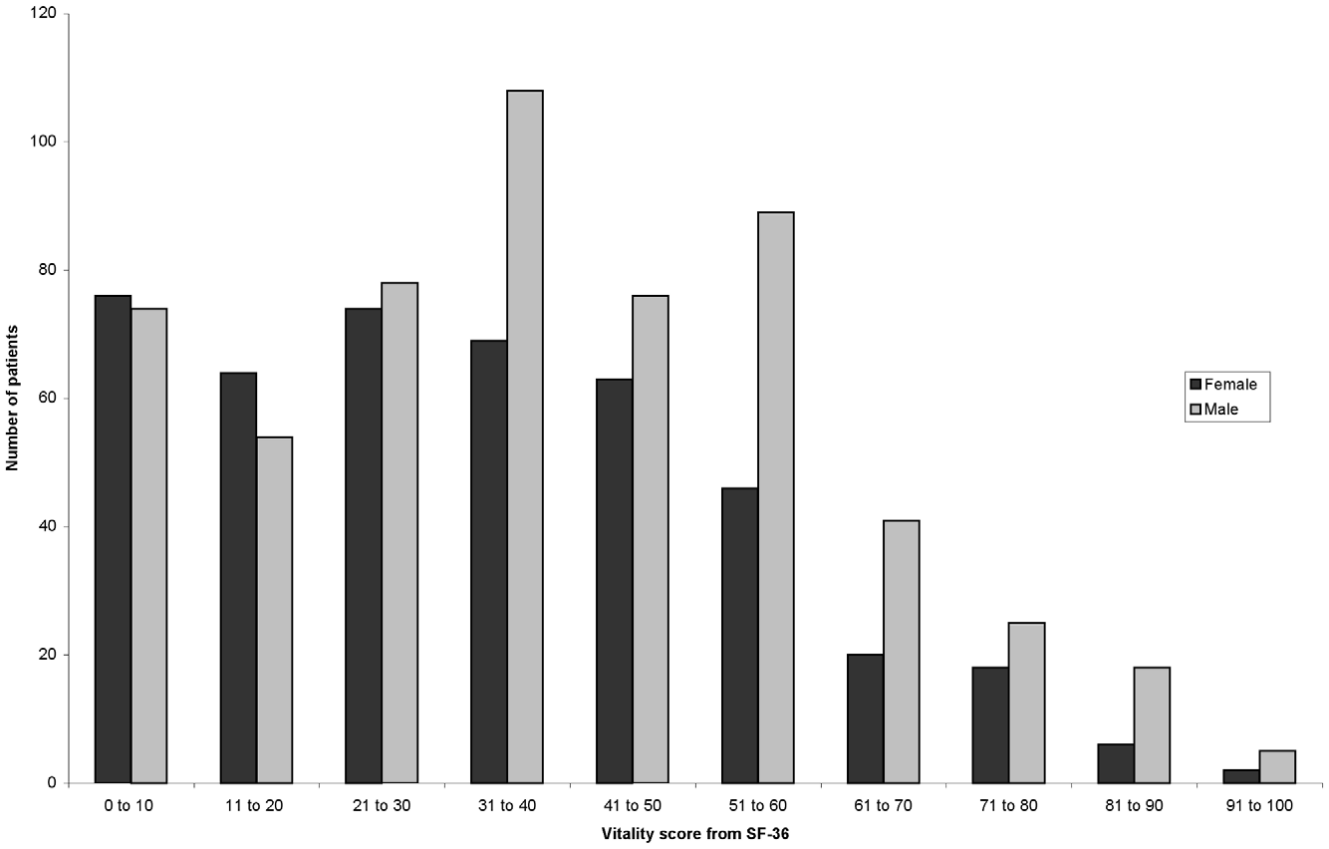
Distribution of SF-36 vitality scores, according to gender. Higher vitality scores indicate less fatigue.

The mean age of the participants was 71.1 (SD 10.8) years and 602 (55.7%) were men. Other demographic details are shown in table 1. Median SF-36 vitality score (interquartile range) was 37.5 (20.0, 55) for the entire group, 40 (IQR 25, 55) for men and 35 (20,50) for women. The distribution of the vitality scores for men and women is shown in figure 1.

**Table 1:** Baseline characteristics of the 1080 participants.

Characteristic		Number	%
Sex	Male	602	55.7
Stroke type	Ischaemic	918	85.0
	Haemorrhagic	82	7.6
	Indeterminate	53	4.9
	Not stroke	27	2.5
Stroke sub-type	LACS	285	26.4
	PACS	446	41.3
	POCS	124	11.5
	TACS	222	20.6
	Other	3	0.28
Infarct visible	Yes	265	24.5
Atrial fibrillation	Yes	166	15.4
Systolic blood pressure	<140	200	18.5
	140 - 159	306	28.3
	160 - 179	273	25.3
	> = 179	301	27.9

### Baseline predictors of fatigue

We analysed SF-36 data from 1080 patients, of whom 1006 (93%) had a vitality score recorded. The amount of variability in the vitality score explained by the variables we included was very small [adjusted R^2^ values for the 4 models were: 0.037, 0.054, 0.004, 0.034 respectively].

Three of the four final multiple regression models contained the same variables although the parameter estimates varied slightly. However, when the missing values were set to the maximum recorded value, the model was very different, with only gender predicting fatigue. These equations allow us to estimate SF-36 vitality score if we have data for age, gender, pathological type (ischaemic, haemorrhagic or indeterminate), and subtype (TACS, PACS, LACS, POCS or ‘other’ stroke subtype).

Each of models is specified in full below, according to how missing data were dealt with:

- Missing values excluded

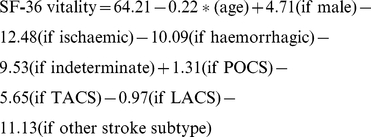

- Missing values set to minimum

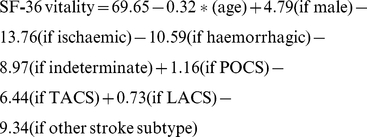

- Missing values set to maximum

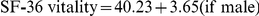

- Missing values imputed at mean

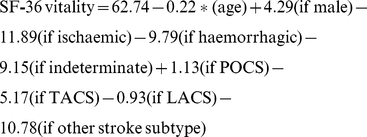

Note: ‘Other’ stroke syndrome is defined as those individuals whose clinical syndromes could not be assigned to one of the four OCSP syndromes.

### Association between mood and fatigue

**Figure 2:**
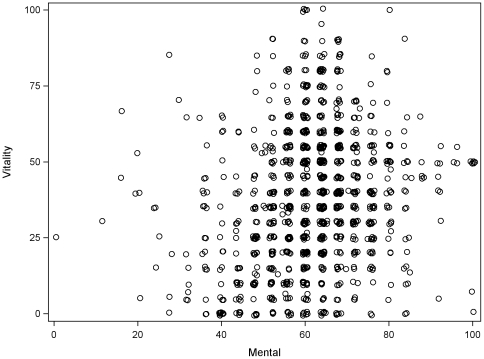
Relationship between SF-36 mental health and vitality. Higher scores indicate better health status. As the number of data points are limited, a small random component (‘jitter’) has been added to each variable to better demonstrate the number of patients at a single co-ordinate. (Pearson correlation coefficient 0.20, p<0.001, n = 1004).

**Figure 3:**
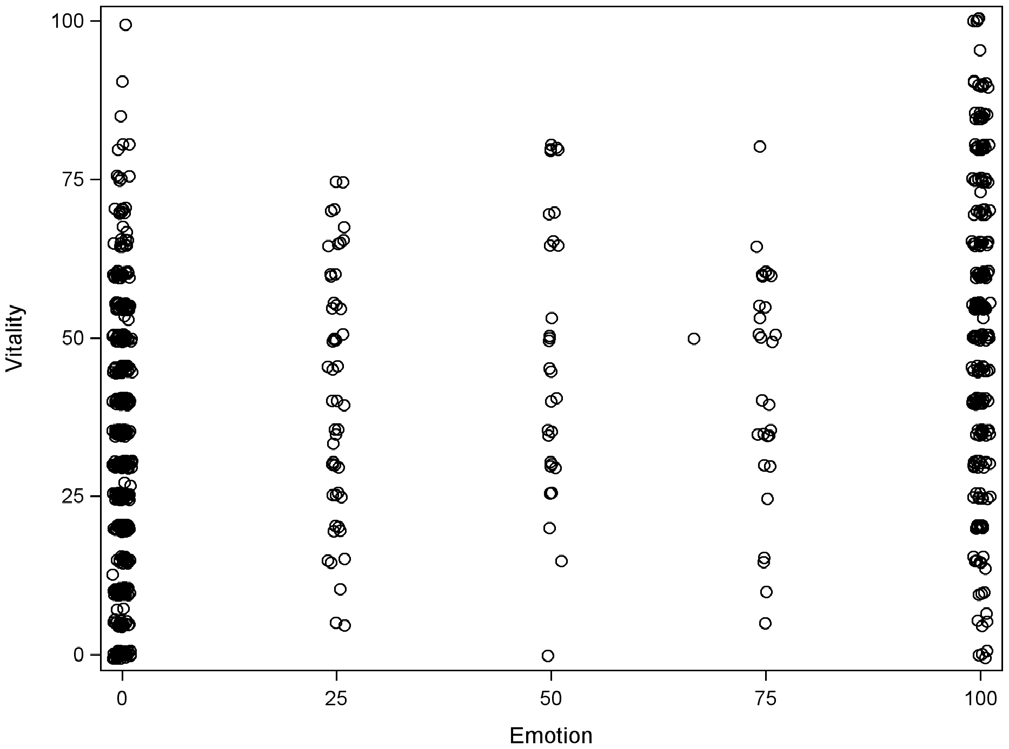
Relationship between SF-36 role emotional and vitality. Higher scores indicate better health status. As the number of data points are limited, a small random component (‘jitter’) has been added to each variable to better demonstrate the number of patients at a single co-ordinate. (Pearson correlation coefficient 0.38, p<0.001, n = 863).

We determined association between mood and fatigue, using SF-36 mental health scores, SF-36 emotional role scores and vitality scores. There were missing data from 6.7% of mental health scores and 18.3% of emotional role scores. Figure 2 shows the univariate relationship between SF-36 mental health and vitality (Pearson correlation coefficient 0.20, p<0.001, n = 1004). Figure 3 shows the univariate relationship between SF-36 role emotional and vitality (Pearson correlation coefficient 0.38, p<0.001, n = 863).

For the multiple logistical regression models, gender, age, mental health score and emotional role function were significant predictors of vitality in all four models. The amount of variability in the vitality score explained by variation in the models is small [adjusted R^2^ for the 4 models are: 0.18, 0.29, 0.27, 0.16 respectively]. However, these models explain much more of the variance than those models which did not include mental health and emotional role scores.

The regression equations for all 4 models are shown below.

- Missing values excluded

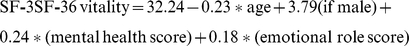

- Missing values set to minimum

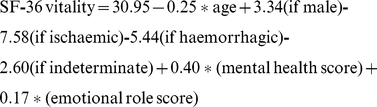

- Missing values set to maximum

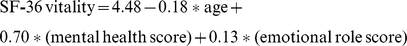

- Missing values imputed at mean

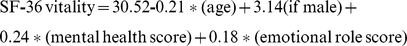
(‘*’ means multiply)

### Influence of fatigue on subsequent survival

Survival data to November 2000 were available for 1072/1080 (99.3%) participants, of whom 420/1072 (39.2%) had died. The median number of days' follow-up from randomisation to November 2000 was 5.9 years (IQR 3.8 to 6.6). The median length of follow-up from when SF-36 was measured, in the 1016 participants with this information recorded, was 5.2 years (IQR 2.4 to 5.3).

Table 2 presents multivariate analyses showing independent predictors of survival. Increasing age, atrial fibrillation (at randomisation) and ‘other’ stroke subtype predicted shorter survival in all four models. In two models, higher SF-36 vitality at follow-up predicted longer subsequent survival.

**Table 2:** Multivariate analyses showing independent predictors of survival, according to the method of dealing with missing SF-36 data.

Parameter	Hazard ratio	Lower 95% CI	Upper 95% CI
**Missing data set to mean**			
Age	1.060	1.048	1.072
LACS	0.78	0.585	1.039
‘Other’ stroke type	4.435	1.083	18.169
PACS	0.875	0.676	1.132
POCS	0.68	0.466	0.992
Atrial fibrillation (at randomisation)	0.708	0.555	0.904
SF-36 vitality	0.993	0.987	0.999
SF-36 social role	0.996	0.992	1.000
**Missing data set to maximum**			
Age	1.061	1.049	1.073
LACS	0.778	0.584	1.036
‘Other’ stroke type	4.334	1.057	17.767
PACS	0.856	0.662	1.107
POCS	0.669	0.458	0.975
Atrial fibrillation (at randomisation)	0.694	0.544	0.885
SF-36 social role	0.994	0.991	0.997
SF-36 vitality	1.001	0.996	1.005
**Missing data set to minimum**			
Age	1.058	1.046	1.070
LACS	0.784	0.589	1.044
‘Other’ stroke type	4.662	1.139	19.084
PACS	0.873	0.675	1.129
POCS	0.677	0.464	0.988
Atrial fibrillation (at randomisation)	0.710	0.556	0.905
SF-36 vitality	0.988	0.984	0.993
**Missing data excluded**			
Age	1.065	1.052	1.078
Atrial fibrillation (at randomisation)	0.689	0.530	0.897
SF-36 general health	0.989	0.984	0.994
SF-36 vitality	0.994	0.988	1.001

LACS = lacunar syndrome, PACS = partial anterior circulation syndrome, TACS = total anterior circulation syndrome, POCS = posterior circulation syndrome

Note that hazard ratios and 95% CI for SF-36 vitality are provided for all four models, even though the hazard ratio reached statistical significance in only two of the models.

## Discussion

To our knowledge, this is the first study to explore not only the factors at stroke onset that predict post-stroke fatigue, but also the relationship between fatigue and mood, and the influence of fatigue at follow-up on subsequent survival. Increasing age, worse emotional role function and worse mental health score measured at follow-up were significantly associated with fatigue. Fatigue on follow-up was associated with significantly reduced subsequent survival in two models. Although we did not specifically aim to compare SF-36 vitality scores after stroke with a general population, we noted that the median SF-36 vitality score of 37.5 was substantially lower than the mean SF-36 vitality score of 55.8 from the general population aged 70-74 years [20].

The study had several strengths: it was a very large, prospective and conducted in a single, well characterised cohort of stroke patients recruited at a uniform time point (stroke onset); it used a valid and reliable measure of fatigue after stroke (SF-36 vitality score) [11], [12]. It explored baseline predictors of fatigue, relationship between fatigue and mood, and the influence of fatigue on survival in the same large group of patients.

Increasing age, lower emotional role function and lower mental health score were significantly associated with fatigue in all four models and female sex was also significant in three models. Importantly, the factors we identified accounted for only a small amount of variance in fatigue, suggesting that other factors, for which we did not collect data, must also be associated with fatigue.

Previous cross-sectional studies have explored the relationship between fatigue and mood disorders after stroke, with most but not all finding associations. Thus, the evidence points to an association between fatigue and mood disorders. However, we cannot determine direction of causality from current evidence i.e. whether fatigue causes mood disorders or whether mood disorders cause fatigue. In the general population, SF-36 vitality scores are lower in women than men, and fall with increasing age [20], and fatigue is a well-recognised symptom of mood disorders. Thus, these findings are consistent with findings from the general population.

The extent to which the post-stroke vitality scores might have been explained by pre-stroke scores is uncertain. One previous study found that 62% of patients with post-stroke fatigue had had pre-stroke fatigue [4], suggesting that some fatigue was present *before* the stroke and that some develops *after* the stroke.

After correction for role emotional and mental health, we found no evidence of a relationship between OCSP stroke subtype, as an indicator of stroke lesion location and size, and fatigue. Ours is the largest study to date to explore associations between fatigue and stroke lesion location and size, and so this is an important negative finding. Two previous studies have suggested that fatigue is more common in brain stem strokes [5], [7], although other studies did not find an association between brain stem strokes and fatigue. Although fatigue may not be related to lesion location, it is, nevertheless, possible that fatigue may have a ‘central’ origin i.e. it might be a direct consequence of a cerebral infarct or cerebral haemorrhage [21]. The observation that fatigue after stroke is unlike anything ever experienced before by stroke survivors [22] supports the idea that fatigue might be directly caused by the brain lesion.

One study has suggested that fatigue may be related to dysregulation of blood pressure after stroke, perhaps as a result of antihypertensive drugs [10]. We did not find an association between *baseline* blood pressure and fatigue, but were unable to explore relationship between fatigue and *current* blood pressure.

Intriguingly, lower SF-36 vitality scores (i.e. more fatigue) in our study were associated with shorter survival. However, after correction for pain, social role functioning, physical function and general health, fatigue remained a significant predictor in only two models. Nonetheless, if confirmed, this would strengthen the case for further studies into the biology of post-stroke fatigue.

Our study has some weaknesses. Our data were from a randomised trial rather than from a population-based study, although the inclusion criteria for IST were broad. The SF-36 scores were obtained at a median of 64 weeks after stroke onset which perhaps somewhat limits the generalisability of the findings. The 77% response rate leads to some degree of uncertainty in the analyses, though it is similar to the previous postal survey of fatigue after stroke [1]. There were missing data items in some of the SF-36 forms. We could have simplified data analysis by performing only one analysis after excluding those with missing data, but this might have biased our results, because non-responders may have been systematically different from those who did respond e.g. more depressed, more fatigued. There is no consensus about how to deal with missing data in this type of study. Each possible method has its own potential biases, so we dealt with these missing data items in four different ways statistically. Reassuringly, final regression models for SF-36 vitality were similar, and importantly, all explained only a small amount of variance in fatigue. We were unable to correct our analyses for levels of dependence, which is known to influence survival [18], but we did correct for SF-36 self-reported physical function. There were a few patients who did not have a definite diagnosis of stroke; we decided to include these as the physician recruiting them to IST felt that they had had a stroke. Although fatigue is a complex experience for patients, in large studies such as this, it is necessary to use a quick, simple tool, such as the SF-36 vitality component, that has been validated against a case definition for clinically significant fatigue after stroke [11], [12]. Despite these weaknesses, this very large study represents an important new contribution to the sparse literature on fatigue after stroke.

What are the implications of this study? Physicians should consider mood disorders in patients with post-stroke fatigue. Further research is required to determine the relationship between pre-stroke and post-stroke fatigue, the natural history of fatigue after stroke, the direction of causality between mood disorders and fatigue and to determine whether fatigue is an independent predictor of survival and, if it is, the biological mechanism that accounts for the survival disadvantage that fatigue confers. There is clearly also a need to develop interventions for fatigue after stroke and test them in appropriate trials [23].
